# Postoperative Atrial Fibrillation Reduced by Intraoperative and Postoperative Cell Saver System in Coronary Artery Bypass Graft Surgery

**DOI:** 10.5152/TJAR.2022.21121

**Published:** 2022-06-01

**Authors:** Muharrem Koçyiğit, Özgen Ilgaz Koçyiğit, Ahmet Ümit Güllü, Şahin Şenay, Cem Alhan

**Affiliations:** 1 Department of Anaesthesiology, Acıbadem Mehmet Ali Aydınlar University Vocational School of Health Services, İstanbul, Turkey; 2 Department of Cardiovascular Surgery, Acıbadem Mehmet Ali Aydınlar University Faculty of Medicine, İstanbul, Turkey

**Keywords:** Blood transfusion, cell salvage, coronary artery bypass graft surgery, postoperative atrial fibrillation

## Abstract

**Objective:**

Postoperative atrial fibrillation is commonly seen after cardiac surgery. One of the contributing factors is mediastinal shed blood and inflammation. Cell salvage techniques can reduce allogenic blood transfusion and reduce inflammation. The aim of this study was to investigate the reduction of postoperative atrial fibrillation by using the cell-salvage system.

**Methods:**

Patients who underwent isolated coronary artery bypass graft surgery (n = 498) were analyzed retrospectively in 2 groups. Postoperative atrial fibrillation group (n = 75) and non-postoperative atrial fibrillation group (n = 423). Preoperative and postoperative demographic and clinical data were compared between the 2 groups, respectively. Postoperative atrial fibrillation and possible contributing factors were analyzed with multinomial logistic regression analysis.

**Results:**

In the postoperative atrial fibrillation group, the patients’ age and European System for Cardiac Operative Risk Evaluation (Euroscore)were higher than in the non-postoperative atrial fibrillation group (*P * = .001 and *P *= .003, respectively). Postoperative intensive care unit stay and hospital stay were longer in the postoperative atrial fibrillation group than in the non-postoperative atrial fibrillation group (*P  *= .001 and *P*  = .046, respectively). There were no statistical differences in mortality between groups. The incidence of postoperative atrial fibrillation decreased with the use of cell saver system and low Euroscore.

**Conclusion:**

The use of a cell salvage device intraoperatively and during the early postoperative period can decrease the incidence of postoperative atrial fibrillation group.

**Main Points**

- Cell saver devices can reduce blood transfusion and inflammation.
- The causes of postoperative atrial fibrillation are multifactorial, including patient characteristics, predisposing factors, types of surgical procedure, mediastinal shed blood, and systemic inflammation.
- Lower Euroscore and use of cell-saver system reduce the incidence of postoperative atrial fibrillation.

## Introduction

Postoperative atrial fibrillation (POAF) is seen in 19%-50% of patients who undergo cardiac surgery and tends to occur within the first 5 days after the surgery.^1,2^ Postoperative atrial fibrillation is associated with increased postoperative morbidity, longer hospital stays, and higher mortality rates.^1,3^ The development of POAF is considered to be multifactorial, with contributing factors including patient characteristics, predisposing factors, types of surgical procedure, and intraoperative factors, such as mediastinal shed blood and systemic inflammation.^3,4^

Cell salvage can reduce the need for blood transfusion and the related risks of infectious and non-infectious complications. Cell salvage can increase erythrocyte viability, maintain the disc shape, and improve the tissue oxygen delivery.^5^ It can also reduce the inflammatory response to surgery.^5,6^

In this retrospective study, we wanted to identify whether there is a relationship between the use of the cell rescue system during and after the operation and the incidence of POAF.

## Methods

After receiving institutional review board approval for the study, patients’ data were retrieved from the electronic medical records and database of our institution. The requirement for individual informed consent was waived because of the retrospective nature of the study.

Patients who underwent isolated coronary artery bypass graft (CABG) surgery between April 1, 2014, and August 31, 2019, were included in the study. Patients who underwent robotic surgery, off-pump surgery, concomitant surgery, valve surgeries, major vessels surgeries, and who had previous cardiac surgery were excluded. Patients with preoperative atrial fibrillation (AF) were also not included in the study.

Clopidogrel or other oral anticoagulant agents (except aspirin), as well as oral anticoagulants, have been stopped at least 3 days before the procedure.

Magnesium sulfate 6 mmol has been infused the day before the surgery and continued per a day for 4 days as our clinical practise.^7^

All operations were performed by the same surgical team and anaesthesiologist as in clinical practice. Tranexamic acid (25 mg kg^−1^, intravenously [i.v.]) has been infused into all patients with induction of anaesthesia. During cardiopulmonary bypass (CPB), blood from cardiotomy suction was transfused continuously without cell salvage. Red blood cells were transfused if the hematocrit value fell below 17% during CPB or below 20% after CPB.

In patients in whom the cell-saver system was used during the operation, blood was collected throughout the whole cardiac procedure from the pericardial and pleural spaces into the collection reservoir of the cell salvage device (Cell saver-XTRA®; Sorin, London, UK). After removal of the aortic and venous cannulas, protamine was administered i.v. to all patients. All remaining pump contents were washed before being returned to the patients by cell salvage. After the closure of the sternum, chest drainage tubes were connected to an autotransfusion circuit in a sterile plastic bag and transfused to the patients continuously during the intraoperative period.

In patients in whom the cell-saver system was not used during the operation, conventional cardiotomy suction was used, and the residual blood from the heart–lung machine was directly packed and re-transfused to the patient through a standard blood transfusion set.

The technical specifications, application, and advantages of the system were informed to all the patients. Cell-saver system set up was used in patients whose private health insurance covers this kind of systems and financial supports.

In all patients, hemodynamic monitoring, ventilation, and postoperative analgesia were managed using a standard clinical protocol. Autotransfusion was continued in the intensive care unit (ICU) for 6 hours postoperatively in whom the cell-saver system was used to drainage the chest and mediastinal tubes. Chest tube output was not taken into account during this period.

After discharge from ICU, all patients were monitored with alarm-triggered 7-lead telemetry system.

Demographic data, Euroscore, ejection fractions (EF) (%), hematocrits, creatinine levels, use of medicines, and the existence of diabetes mellitus and hypertension were reviewed preoperatively.

In addition to the cross-clamp and CPB durations during surgery, the duration of endotracheal intubation and ICU stay, creatinine levels at discharge, chest tube drainage volume, and rates of postoperative stroke, new onset of dialysis, blood transfusion, and mortality were recorded postoperatively.

The primary outcome was the morbidity and mortality between the POAF and non-POAF groups. The secondary outcome was the correlation between POAF and predictive factors.

### Statistical Analysis

Data are reported as percentages or as means ± standard deviations. Univariate comparisons were made using the *χ* ^2^ test or Fisher’s exact test for categorical variables, and the *t*-test for continuous variables. Variables with a *P*-value of .1 or less were entered into the logistic regression analysis. A multinomial logistic regression analysis was used to examine the relationships between POAF and possible contributing factors. *P *< .05 were considered to be significant.

## Results

In total, there were 498 patients who underwent isolated CABG surgery in 62 months period. Cell salvage was used in 152 patients.

The patients in the POAF group were significantly older than those in the non-POAF group. Euroscore levels were significantly higher in the POAF group than in the non-POAF group. There was no statistically significant difference in the other preoperative and demographic values between the groups (Table 1). The ICU and hospital stay were longer in the POAF group than in the non-POAF group (Table 1). In the POAF group, 10 patients had a blood transfusion in whom the cell-saver system was not used. In the non-POAF group, 41 patients had a blood transfusion among whom the cell-saver system was used in 8 patients. The other 134 patients who used the cell-saver system did not receive blood transfusion. Postoperative pulmonary complications were higher in the POAF group than in the non-POAF group (Table 1). There were no differences in mortality. The overall mortality rate was 0.8%.

In the regression analysis, POAF has an inverse relationship with the cell-saver system (Table 2).

## Discussion

In this retrospective study, the incidence of POAF was found to be significantly reduced with the use of cell salvage in patients undergoing CABG.

Postoperative atrial fibrillation is a common postoperative arrhythmia in patients undergoing isolated CABG.^2^ Several perioperative factors, including advanced age, higher Euroscore, hypertension, diabetes mellitus, chronic obstructive pulmonary disease, hyperthyroidism, heart failure, low EF, obesity, smoking, atrial enlargement, myocardial infarction, valve disease, surgical atrial injury, volume status, inotropic agents, and withdrawal of angiotensin-converting enzyme inhibitors and beta-blockers contribute to POAF.^2,3,8^ The occurrence of POAF may increase the length of hospital stay, morbidity, and mortality.^3,9^

In our study, patients with POAF were older and had higher Euroscore than the patients without POAF. The ICU and hospital stay were longer in patients with POAF in agreement with previous studies.^1,10^ In regression analysis, age >70 years, EF <30%, and non-elective surgery were not statistically significant for POAF. Euroscore <5 and using the cell-saver system were statistically reducing POAF.

Oxidative stress and systemic inflammation also contribute to the development of POAF in patients who have undergone cardiac surgery.^4,11^ Perioperative blood transfusion has been reported to cause systemic inflammatory changes; therefore, an increase in the AF rate may be expected in patients transfused with blood during cardiac surgery.^4,11^ Alameddine et al^12^ stated that the risk of POAF increased with the higher number of transfusions. On the other hand, Vlahou et al^13^ reported that the blood products transfusions in CABG surgery were not associated with the increased risk of POAF. In our study, there were no statistically significant differences in the rate of transfusions between the groups.

Postoperative pulmonary complications were seen in 2 patients in the POAF group in which one of them had permanent neurologic deficit causing longer mechanical ventilation and the other one had pulmonary bleeding with pneumonia.

Cell salvage devices reduce the intraoperative and postoperative blood transfusion requirements in cardiac surgery. In a meta-analysis that included 2282 patients undergoing cardiac surgery, the use of an intraoperative cell saver device reduced the rate of exposure to any allogeneic blood product and red blood cells.^14^ Additionally, there were no difference in hospital mortality, stroke, atrial fibrillation, renal dysfunction, or infection between cell-saver and non-cell saver groups.^14^ In our study, the incidence of POAF was decreased with the use of the cell-saver system.

Pericardial shed blood can activate a coagulation cascade, and a pericardial pro-oxidative, proinflammatory milieu can trigger AF during cardiac surgery.^4,11^

Therefore, strategies to prevent the accumulation of shed blood around the heart can reduce AF. Chest and mediastinal tubes, used to drain shed blood, can become occluded easily because of stasis, and this type of occlusion is seen in about 1 in 3 patients who undergo cardiac surgery.^4,15^ Attempts, including stripping, squeezing, milking, and open suction of chest tubes, have been made to maintain chest tube patency and prevent shed blood. These techniques can be ineffective and harmful in some patients.^15^ The use of a cell-saver system for 6 hours postoperatively may decrease mediastinal shed blood by suctioning blood continuously. Therefore, this technique may also prevent pericardial accumulation of blood.

Centrifugation and washing of shed blood using a cell-saver system reduce the amounts of microparticulates and activated proteins in autologous blood before retransfusion.^15^ In this way, the red blood cells become concentrated, and debris and damaged red blood cells are removed,^16^ which reduces the severity of systemic inflammation.

Cell-saver systems reduce the amount of inflammatory mediators, such as cytokines, and neutrophilic proteases.^17^ It was also found to reduce systemic levels of the proinflammatory markers interleukin-6 (IL) and IL-8 at 6 hours after CPB.^18^ Therefore, a decrease in the rate of POAF is expected in patients in which cell salvage is used.

The main limitations of our study were the non-randomized, observartional study with relatively small group. One of the strengths of the study was that the patient sample was from a single center, and all operations were done by the same surgeons and anaesthesiologists using similar practices for blood transfusion. This study was a retrospective study, but the database was well-defined. In the future, randomized-controlled trials can be powerful in demonstrating the effect of cell-saver system on POAF.

To our knowledge, this study is the first to determine the effect of a cell-salvage technique on the development of POAF after CABG. In this retrospective study, we showed that the incidence of POAF decreased by 3.5 times with use of the cell-salvage system. This observation is clinically important and may be the result of the decreased need for blood transfusion and reduction of systemic inflammation by washing out inflammatory mediators and lipids from shed blood with the cell-salvage device.

The use of a cell-salvage device intraoperatively and during the early postoperative period can decrease the incidence of POAF after cardiac surgery.

## Figures and Tables

**Table 1.** Patients’ Demographics and Clinical Data

	Non-POAF (n = 423)	POAF (n = 75)	*P*
Age (years)	61.70 ± 9.94	65.96 ± 8.40	.001
Sex (female/male) (n)	64/359	10/65	.687
Height (cm)	168.24 ± 10.49	169.72 ± 12.87	.604
Weight (kg)	82.09 ± 13.91	81.06 ± 14.15	.559
BSA	1.98 ± 0.18	1.97 ± .020	.686
Euroscore (%)	3.42 ± 3.12	4.61 ± 3.24	.003
NYHA classification (%)			
NYHA1	34.5% (n = 146)	34.6% (n = 26)	.202
NYHA2	58.4% (n = 247)	53.3% (n = 40)	
NYHA3	5.9% (n = 25)	12.0% (n = 9)	
NYHA4	1.1% (n = 5)	0.0% (n = 0)
Left ventricle EF (%)			
>50%	74.7% (n = 316)	64.0% (n = 48)	.152
30%-50%	24.1% (n = 102)	34.7% (n = 26)	
<30%	1.2% (n = 5)	1.3% (n = 1)
Hypertension (%)	66.7% (n = 282)	73.3% (n = 55)	.255
Diabetes mellitus (%)	42.3% (n = 179)	40.0% (n = 30)	.708
Hypercholesterolemia (%)	57.9% (n = 245)	58.7% (n = 44)	.904
COPD (%)	3.8% (n = 16)	4.0% (n = 3)	.928
Smoking (%)			
Smokers	31.2% (n = 132)	34.7% (n = 26)	.153
Former smokers	36.4% (n = 154)	44.0% (n = 33)	
Never smoked	32.4% (n = 137)	21.3% (n = 16)
Medications			
Beta blockers (%)	53.9% (n = 228)	42.7% (n = 32)	.073
ACE inhibitors (%)	37.4% (n = 158)	42.7% (n = 32)	.160
Aspirin (%)	73.5% (n = 311)	76.0% (n = 57)	.653
Lipid lowering agents (%)	40.0% (n = 169)	34.7% (n = 26)	.387
Elective surgery (%)	75.2% (n = 318)	70.7% (n = 53)	.170
Preoperative hematocrit level (%)	40.66 ± 4.29	39.79 ± 4.02	.107
CC time (min)	59.56 ± 19.65	56.98 ± 18.63	.299
CPB time (min)	94.87 ± 28.51	93.87 ± 26.06	.779
Number of distal anastomoses	3.48 ± 0.92	3.44 ± 0.75	.793
**Postoperative data**			
Intubation time (h)	6.88 ± 7.04	7.78 ± 5.73	.298
Chest tube output (mL)	477.38 ± 316.76	471.26 ± 195.36	.871
Transfusions			
RBC transfusion (%)	9.7% (n = 41)	13.3% (n = 10)	.338
FFP transfusion (%)	5.2% (n = 22)	8.0% (n = 6)	.332
Plasma transfusion (%)	2.1% (n = 9)	0% (n = 0)	.202
ICU duration (h)	19.80 ± 8.39	25.77 ± 32.77	.001
Cell-saver usage (%)	33.6% (n = 142)	13.3% (n = 10)	<.001
New-onset stroke (%)			
Transient	0.2 % (n = 1)	0	.054
Permanent	0	1.2% (n = 1)
New-onset dialysis (%)			
Transient	0	0	.388
Permanent	0.5% (n = 2)	1.3% (n = 1)
Pulmonary complications (%)	0	2.7 % (n = 2)	.023
Sternal dehiscence (%)	0	1.3% (n = 1)	.151
Overall infections (%)	0.4% (n = 2)	0	.720
Discharge hematocrit level (%)	29.04 ± 4.23	29.31 ± 3.85	.616
Hospital duration (days)	6.59 ± 3.79	7.58 ± 4.89	.046
ICU readmission (%)	2.6 % (n = 11)	0	.158
Hospital readmission (%)	0.7% (n = 3)	1.3% (n = 1)	.481
Mortality (%)	0.5% (n = 2)	2.7% (n = 2)	.110

POAF, postoperative atrial fibrillation; BSA, body surface area; NYHA, New York heart association; EF, ejection fraction; COPD, chronic obstructive pulmonary disease; ACE, angiotensin-converting enzyme; CC, cross clamp; CPB, cardiopulmonary bypass; RBC, red blood cell; FFP, fresh frozen plasma; ICU, intensive care unit.

**Table 2.** Logistic Regression Analysis of AF

	**Adjusted Odds Ratio**	**95% CI**	*P*
Age >70 (years)	1.229	0.645-2.345	.534
Non-elective surgery	1.017	0.516-2.008	.960
EF <30%	1.391	0.288-6.712	.671
Euroscore ≥5	2.092	1.018-4.298	.045
Without cell-saver system	3.516	1.690-7.316	<.001

EF, ejection fraction.
